# Practical accounting for eye programmes: an introduction

**Published:** 2013

**Authors:** Heiko Philippin, Richard Hess

**Affiliations:** Head of postgraduate training and glaucoma specialist: Kilimanjaro Christian Medical Centre, Moshi, Tanzania. **philippin@gmx.de**; Management consultant, Singen, Germany. **richardhess09@gmail.com**

**Figure:**
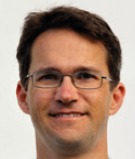
Heiko Philippin

**Figure:**
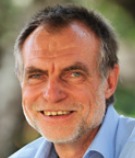
Richard Hess

## No institution can survive without balanced books

Financial and administrative management are fundamental to any institution. This applies also to eye care projects. Eye care managers benefit from a solid understanding of accounting and financial management as it will help them to work in, or supervise, this important area.

## Basic accounting

It is not possible to provide a comprehensive introduction to accounting in this article. A few definitions might, however, be helpful as a starting point for further reading.

- Budgeting is an essential process and should always happen when a new activity is planned. A **budget** is an estimate of future expenditure and income. Each activity needs its own budget. The budget is what you check your income and expenditure against when running the activity, to make sure you have enough funds and are not spending too much.
- **Cost centres** are different departments or activities. Examples of cost centres include the outpatient department, the operating theatre, and outreach. A cost centre will have its own account. It is useful to consider the cost centre's total expenses and total income to see whether it is running according to budget.
  **Figure:**
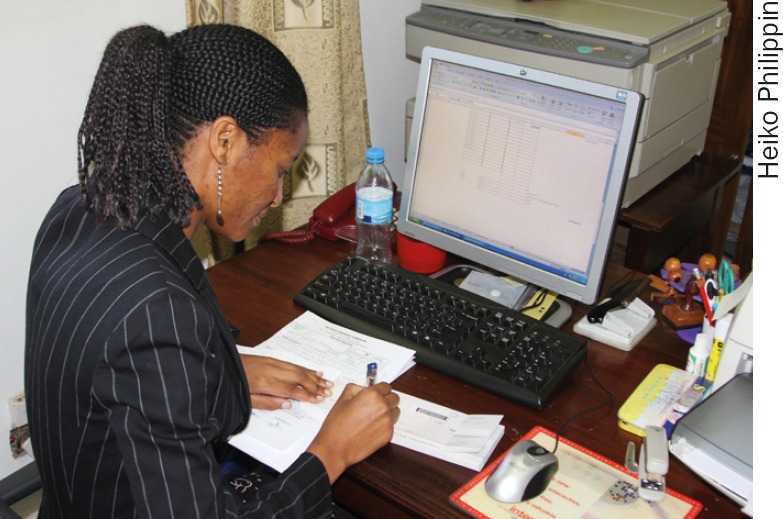
Spreadsheet software can be a helpful accounting tool.
- An **account** is a record of financial transactions, including **payments** (expenses) or **deposits** (income). Each transaction is specified by date, type (cash, cheque, etc.) and detail, such as purpose of a payment, e.g. fees for surgery. Typical examples of accounts include petty cash, bank accounts or stock accounts. It makes financial management easier if you set up different accounts for different activities. The listing of the account names is called the **chart of accounts**.
- **Double entry:** whatever is taken from one account has to be received by another account. For example if salaries are paid, expenses increase in the salaries account and at the same time funds decrease in the bank account.
- A **ledger** is a book or computer file where all monetary transactions for each account are recorded. Transactions must be approved and accompanied by receipts.

## Software solutions

Initially, systems and standard procedures can be developed manually (on paper), also taking into consideration the country's legal requirements. Spreadsheet software is good for keeping records of income and expenditure. Once these standard procedures are established, they can be translated into a software system for easier and safer data entry, planning and reporting. A general accounting software package is often good enough, as you can use a special chart of accounts that reflects the needs of the project. Some examples are Quickbooks, GnuCash or WebERP. Quickbooks is commonly used in companies and NGO projects; details can be found at www.quickbooks.intuit.com. GnuCash is an example of an open source and free software system which runs on all platforms (**http://www.gnucash.org/**). WebERP is also free and is a large system which runs on a server in a network (**http://www.weberp.org**).

**Accounting challenges in donor-funded projects**

**1 Keeping funds separate – and reporting accurately**

Different donors (or partners) will require different financial reports about the activities they fund – often at different times.

It can be a challenge in eye care programme management to keep track of spending and to generate different reports for different donors with just a general income and expenditure statement.

A practical solution is to post donor funds, after they have been received, into a **liability account,** with sub-accounts for each donor. A liability account tracks how much a person or business (in this case, the eye care programme) owes a creditor, in this case the donor. The liability account tracks debts to the donor (i.e. the services to be delivered).

A statement of expenditure on respective activities should be issued once a month. These expenses can be posted from the relevant sub-account in the liability account to the income account, meaning that a debit is assigned to the liability account and the credit is offset to the income account.

With this approach, the donor liability account always shows the current state of different donor funds. If necessary, a report can be consolidated either in the general account or in a supporting document. [Note: receiving donor funds is **not** yet an income since these funds are designated for specific purposes and become an income only when spent.]

**Figure:**
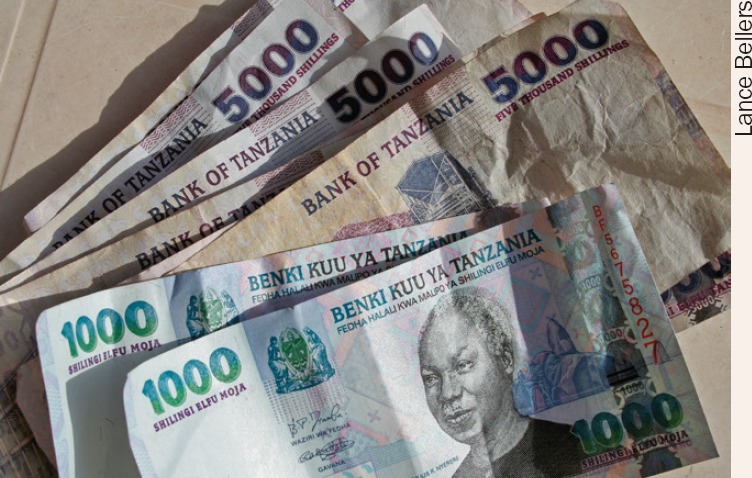

**2 Dealing with different currencies**

For different currencies, different accounts must be used. If average exchange rates are used internally, reporting becomes complicated because expenses vary over time according to changing exchange rates. A solution could be to use only one ‘reporting’ currency in agreement with the donors.
